# Interconnected clusters of invasive meningococcal disease due to *Neisseria meningitidis* serogroup C ST-11 (cc11), involving bisexuals and men who have sex with men, with discos and gay-venues hotspots of transmission, Tuscany, Italy, 2015 to 2016

**DOI:** 10.2807/1560-7917.ES.2018.23.34.1700636

**Published:** 2018-08-23

**Authors:** Alessandro Miglietta, Cecilia Fazio, Arianna Neri, Patrizio Pezzotti, Francesco Innocenti, Chiara Azzari, Gian Maria Rossolini, Maria Moriondo, Francesco Nieddu, Stefania Iannazzo, Fortunato D’Ancona, Francesco Paolo Maraglino, Raniero Guerra, Giovanni Rezza, Fabio Voller, Paola Stefanelli

**Affiliations:** 1 Regional Health Agency of Tuscany, Epidemiologic Observatory, Florence, Italy; 2 Department of Infectious Diseases, Istituto Superiore di Sanità, Rome, Italy; 3 Units of Epidemiology and Preventive Medicine, Central Tuscany Health Authority, Florence, Italy; 4 These authors contributed equally to this work; 5 Laboratory of Immunology and Infectious Diseases, Anna Meyer Children's University Hospital, University of Florence, Florence, Italy.; 6 Department of Experimental and Clinical Medicine, University of Florence, and Clinical Microbiology and Virology Unit, Florence Careggi University Hospital, Florence, Italy; 7 Ministry of Health, Directorate-General of health prevention, Rome, Italy

**Keywords:** invasive meningococcal disease serogroup C, men who have sex with men (MSM), clusters

## Abstract

In 2015 an increased incidence of invasive meningococcal disease due to serogroup-C (MenC) occurred in Tuscany, Italy. This led the Regional Health Authority of Tuscany to implement a reactive immunisation campaign and to launch an epidemiological field investigation aiming to address targeted immunisation interventions. In 2011–14, 10 MenC cases had been reported compared with 62 cases in 2015–16. The case fatality rate was 21% (n = 13) and 51 cases (82.3%) were confirmed as C:P1.5–1,10–8:F3–6:ST-11(cc11). Overall, 17 clusters were recognised. Six discos and four gay-venues were found to have a role as transmission-hotspots, having been attended by 20 and 14 cases in the 10 days before symptoms onset. Ten and three cases occurred, respectively, among men who have sex with men (MSM) and bisexual individuals, who were involved in 11 clusters. In addition, heterosexual cases (n = 5) attending gay-venues were also found. Secondary cases were not identified. Molecular typing indicated close relationship with MenC clusters recently described among gay, bisexual and other MSM in Europe and the United States, suggesting a possible international spread of the serogroup-C-variant P1.5–1,10–8:F3–6:ST-11(cc11) in this population-group; however, epidemiological links were not identified. In December 2016, a targeted vaccination campaign involving discos and lesbian, gay, bisexual, and transgender (LGBT) associations was implemented. During 2017, 10 cases of MenC occurred, compared with 32 and 30 cases reported in 2015 and 2016 respectively, suggesting the effectiveness of the reactive and targeted immunisation programmes.

## Background

Invasive meningococcal disease (IMD) is a severe, life-threatening consequence of infection with the bacterium *Neisseria meningitidis*, a Gram-negative aerobic diplococcus able to colonise the nasopharynx [1]. *N. meningitidis* can be classified into 13 serogroups, four of which (B,C,W,Y) are most commonly associated to IMD (i.e. septicaemia, meningitis or both) in Europe [2].

In 2014, the European Union (EU) IMD notification rate was 0.5 cases per 100,000 population, with highest rates among infants (10.1/100,000), and serogroup B responsible for 64% of all cases [3].

In Italy, the incidence of IMD is among the lowest in Europe, with a rate of 0.3 per 100,000 in 2015 [4]; but this rate is probably an underestimation [5].

## Outbreak detection

During the period 2010–15, all the Italians regions showed a stable trend in the IMD incidence rate (IR), with the exception of Tuscany, where an increase of cases due to *N. meningitidis* serogroup C (MenC) with the finetype C:P1.5–1,10–8:F3–6:ST-11(cc11), was reported starting in 2015 [6].

Between 2013 and 2016, clusters due to *N. meningitidis* C:P1.5–1,10–8:F3–6:ST-11(cc11) involving gay, bisexual and other men who have sex with men (MSM) were reported in Europe and the United States (US) [7-9]. In response, targeted immunisation programmes were implemented [10], and the European Centre for Disease Prevention and Control (ECDC) issued a Rapid Risk Assessment [11], recommending EU countries to investigate MenC cases in order to identify groups of MSM at higher risk [11].

Consequently, the Italian Ministry of Health (MoH) alerted the 20 Regional Health Authorities in the country and issued specific recommendations for surveillance, prevention and control of IMD [12].

In Italy, clusters due to *N. meningitidis* C:P1.5–1,10–8:F3–6:ST-11(cc11) had already been described by Stefanelli et al. in 2008 and in 2012 [13,14], but no specific risk factors were identified.

In Tuscany, MenC disease increased from 10 cases during 2011–14 to 32 for the year 2015 alone, when the Region contributed for 49.3% of all MenC cases at Italian level [4,6].

This led the Regional Health Authority of Tuscany (RHAT), with the support of the Italian MoH, to implement a reactive immunisation campaign in May 2015, offering a single dose of the tetravalent (ACWY) meningococcal conjugate vaccine or monovalent meningococcal C conjugate (MCC) vaccine, free of charge, to the all residents in the Tuscany region.

Moreover, with the aim to address targeted immunisation programmes, the RHAT implemented a regional epidemiological field investigation (EFI) taking into account the ECDC alert because of the same MenC-finetype [6,11].

The primary objectives of our outbreak-report were to describe the clusters, the transmission-hotspots and the risk-groups identified. Secondary objectives were to show the genomic profiles of MenC cases (used to identify clusters) and to update on the epidemiological situation since our previous rapid-communication dated 24 March 2016 [6].

## Methods

The case definition of IMD in Italy is based on the 8 August 2012 EU Commission Decision 2012/506/EU [15]. A cluster of IMD was defined as two or more cases due to the same serogroup, with direct or indirect contact, occurring within a time interval of 3 months [16]. Clusters were confirmed by molecular characterisation: finetype, alleles Neis0430, *penA, porB*, fHbp variant and electrophoretic type (ET) [17,18]. To describe clusters due to the same strain, this molecular designation was used to assign a strain-number (1 to 8) to each MenC case.

Close contacts of IMD were defined according to ECDC guidelines (e.g. living in the same household, sharing drinks, intimate kissing-partners, etc.) [19].

In addition, the following operational definitions [16] were used to describe MenC cases:

• A primary case of MenC was defined as one occurring in the absence of previous known close contact with another MenC case.

• A secondary case of MenC was defined as one who occurs among close contacts of a primary case-patient ≥ 24 hours after onset of illness in the primary patient.

• Co-primary MenC cases were defined as two or more cases who occur among a group of close contacts with onset of illness separated by less than 24 hours.

### Epidemiological field investigation

The EFI was conceived by the Istituto Superiore di Sanità (ISS – Italian National Institute of Health) after request of support from the RHAT and was coordinated locally by the Regional Health Agency of Tuscany, with the technical/scientific support of the Central Tuscany Health Authority (CTHA) and ISS. It started in May 2015 and was performed retrospectively (cases occurring before May 2015) and prospectively (cases occurring from May 2015 onward).

Face-to-face interviews of cases and/or their proxies/close contacts were conducted using a standardised questionnaire to collect information on the following known risk factors for MenC identified from the literature [7-9,20-24]: clinical conditions, active/passive smoking; illicit drug use; drink sharing (i.e. drinking from the same glass or bottle); sexual intercourse occurring in the 10 days before symptom onset, and whether this was or not a same-sex intercourse. Moreover, to assess possible links with the lesbian, gay, bisexual, and transgender (LGBT) community, the sexual orientation (e.g. self-identification as gay, etc.) and sexual behaviour (e.g. MSM) were investigated. To identify clusters and transmission hotspots, the places visited in the 10 days before symptom onset were listed. In addition, information about travel abroad of cases and their close contacts in the month before IMD onset was collected to assess possible links with the recent clusters among gay, bisexual and other MSM that occurred in EU/US [7-11].

### Diagnosis and molecular analyses

Clinical and laboratory diagnosis was performed by the hospitals of admission, using culture and/or slide agglutination (cerebrospinal fluid (CSF) antigen kits), with commercial antisera (Remel Europe, Ltd, United Kingdom).

The regional reference laboratory for IMD at the Meyer Children’s University Hospital of Florence identified the serogroup by real-time PCR and performed the molecular characterisation.

The Italian National Reference Laboratory at ISS confirmed the presence of *N. meningitidis* and the serogroup by slide agglutination and/or by PCR, further defining the genomic profile of meningococcal DNAs. Multilocus sequence typing (MLST), PorA, FetA typing, and fHbp variant identification were conducted/defined as described on Neisseria.org [25].

The finetype was identified as follows: capsular group: *porA* (P1). VR1,VR2: *fetA* VR: ST (cc). Moreover, the alleles Neis0430, *penA*, *porB* and the fHbp variant were identified, as well as the electrophoretic type (ET) [17].

In addition, whole genome sequencing (WGS) was performed by ISS using the Illumina MiSeq platform (kit v3, 600 cycles) on the 30 available bacterial isolates received. Genomes were analysed and compared using the BIGSdb Genome Comparator tool implemented within the PubMLST website [26]. The comparison tool employs a ‘gene by gene’ approach comparing arbitrary, sequentially assigned, pre-indexed allele identifiers at each locus [2]. Phylogenetic analysis of the isolates was performed analysing the 1,605 loci defined as the core genome in the PubMLST Neisseria database [26], by the core genome MLST (cgMLST) approach. The resulting distance matrices were visualised as neighbour-net networks, generated by SplitsTree4 (version 4.13.1).

### Data analysis

Epidemiological links between cases were analysed through a social network diagram [27].

IR per 100,000 inhabitants was calculated using the 2015–16 population data of Tuscany from the Italian National Institute of Statistics (ISTAT) [28].

To explore if the investigated risk factors differed by sexual-orientation/behaviour, age group (11–25; 26–40; 41–55; 56–70 years) and sex, we first evaluated, at the univariate level, the association of the characteristics of the cases with absence/presence (dichotomous: 0/1) of the specific risk factor and crude odds ratios (OR) were calculated to measure the magnitude of the association. Risk factors considered were: active smoking; passive smoking; illicit drug use; clinical condition; and whether the case in the 10 days before symptoms onset had sexual intercourses, shared drinks and attended: (i) discos (ii) gay-venues (iii) bar/restaurants.

Because all those who self-identified as gay men reported MSM-behaviour, and those who self-identified as bisexuals and heterosexuals respectively reported a sexual behaviour corresponding to their sexual orientation, we grouped in heterosexuals and MSM/bisexuals, as an independent variable. Variables found associated at a statistically significant level in the univariate analyses (i.e. p < 0.05) were entered into a multivariable logistic regression model.

Cases aged under 10 years (n = 5) and over 70 years (n = 4; which include one man and three women with no sexual-intercourses before symptoms onset) as well as one case who did not participate in the face-to-face interview, were excluded from univariate and multivariable analyses.

Results were expressed as OR and adjusted OR (AOR) with 95% confidence interval (CI), and were interpreted as the odds of having a certain risk-factor given a certain sexual-orientation/behaviour among the MenC cases investigated.

The significance level was set at p < 0.05. STATA 13 was used for data analyses.

### Ethical considerations

The EFI was approved by the Regional Ethic Committee of Tuscany. Cases provided written informed consent to participate in the interview and for the publication.

## Results

### Epidemiological update and microbiological results

Since the Rapid Communication dated 24 March 2016 [6], 19 additional laboratory-confirmed cases of MenC were reported from the RHAT to the Italian National Surveillance System for Invasive Bacterial Disease at ISS, leading to a total of 62 cases in the period between 1 January 2015 and 31 December 2016.

Thirty-two (51.6%) cases were reported in 2015 and 30 (48.4%) in 2016, with an IR during 2015–16 of 0.8 per 100,000. The 2015–16 IR ranged from 0.6 per 100,000 in the municipality of Collesalvetti (Province of Livorno) to 17.8 per 100,000 in the municipality of Montelupo Fiorentino (Province of Florence) (data not shown). The most affected area was located between the provinces of Florence, Prato, and the municipality of Empoli (IR: 8.6 per 100,000). The median age of cases was 28 years (range: 22 months–83 years); 30 cases (48.4%) occurred among men and 32 (51.6%) among women. The main clinical presentation was sepsis (n = 31; 50.0%), followed by sepsis and meningitis (n = 26; 41.9%), and meningitis only (n = 4; 6.5%), whereas one case (1.6%) was characterised by a symptomatic pharyngeal and tonsillar disease with high fever (>38.0 °C axillary). Case fatality rate (CFR) was 21% (n = 13).

**Figure 1:**
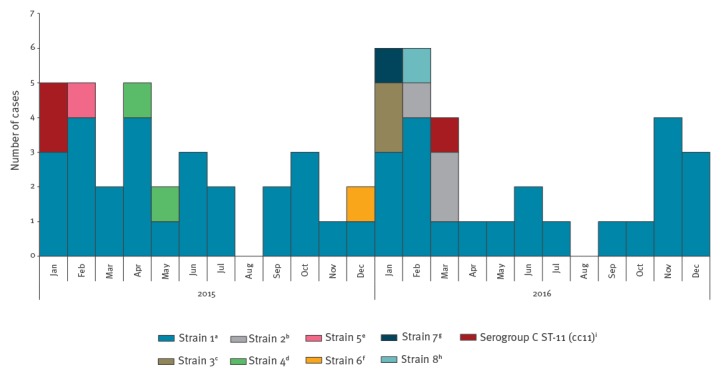
Number of serogroup C meningococcal disease cases by month and molecular characterisation, Tuscany, Italy, 1 January 2015–31 December 2016 (n = 62)

Figure 1 shows the distribution of MenC cases by month and molecular characterisation during 2015–16, and Supplement 1 the molecular characteristics of the *N. meningitidis* affecting each of the 62 MenC cases. The distribution of the cases over the 2 years was similar, following a seasonal pattern of more cases in the winter months of January and February and no cases in August.

Cc: clonal complex; ET: electrophoretic type; ST: sequence type.

Strains are described by capsular group: porA (P1). VR1, VR2: fetA VR: ST (cc) (Neis0430/penA allele) (porB allele) (fHbp variant) (ET).

^a^ Strain 1: C:P1.5-1,10-8:F3-6:ST-11 (cc11)(Neis0430/penA 398/248) (porB 2-2) (fHbp 1.13) (ET-15).

^b^ Strain 2: C:P1.5-1,10-8:F3-6:ST-11/12051 (cc11) (Neis0430/penA 6/7) (porB 2-2) (fHbp 1.808) (ET-15).

^c^ Strain 3: C:P1.5-1,10-1:F3-6:ST-11 (cc11) (Neis0430/penA 398/248) (porB 2-2) (fHbp 1.13) (ET-15).

^d^ Strain 4: C:P1.5-1,10-8:F3-6:ST-2780 (cc11) (Neis0430/penA 398/248) (porB 2-2) (fHbp 1.13) (ET-15).

^e^ Strain 5 C:P1.5-1,10-8:F3-6:ST-11(cc11) (Neis0430/penA 6/7) (porB 2-2) (fHbp allele 669) (ET-15).

^f^ Strain 6: C:P1.7-4,14-3:F3-9:ST-1031 (cc334) (Neis0430/penA 162/599) (porB 2-227) (fHbp 2.19) (ET-15).

^g^ Strain 7: C:P1.5,2:F3-3:ST-11 (cc11) (Neis0430/penA 1/1) (porB 2-2) (fHbp 2.22) (ET-15).

^h^ Strain 8: C:P1.5-1,10-8:F3-6:ST-11936 (cc11) (Neis0430/penA 398/248) (porB 2-2) (fHbp 1.13) (ET-15).

^i^ Incomplete molecular typing due to low DNA concentration in the clinical sample.

Sixty-one cases (98.4%) belonged to the cc-11, 57 of whom (93.4%) were ST-11, two (3.3%) ST-2780, one (1.6%) ST-11936, and one (1.6%) ST-12051. One case (1.6%) was confirmed as cc334/ST-1031.

Overall, 51 cases (82.3%) were confirmed having the finetype C:P1.5–1,10–8:F3–6:ST-11(cc11). The molecular characterisation (finetype, alleles Neis0430, penA, porB, fHbp variant and ET) further identified 48 cases (77.4%) as strain-1, who were distributed throughout the whole biennium 2015–16. Three cases (Supplement 1; cases 43,45,46), who occurred between February and March 2016, were grouped as strain-2. Other MenC cases with different molecular typing were consecutively reported over a short period of time: two, identified as strain-3, occurring in January 2016 (Supplement 1; cases 35,36) and two identified as strain-4 (Supplement 1; cases 17,18) occurring between April and May 2015. Three cases were confirmed as C:ST-11(cc11), but molecular typing remained incomplete due to low DNA concentration in the clinical sample. Four additional cases due to different molecular types were also reported (Supplement 1; cases 7,32,38,41; strain 5 to 8, respectively). 

A total of 1,325 of the 1,605 core genome loci were included in the cgMLST analysis of the 30 bacterial isolates collected; the remaining 280 loci were incompletely assembled.

**Figure 2:**
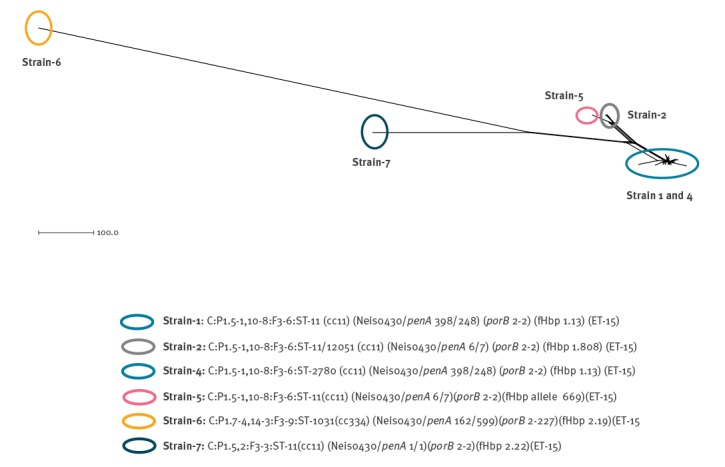
Neighbour-net phylogenetic network of the bacterial isolates collected from meningococcal serogroup C cases, Tuscany, Italy, 2015–2016 (n = 30)

Figure 2 shows a main tight clustering group (mean distance of 26 loci), that comprises 22 isolates belonging to strain-1, and two isolates belonging to strain-4. The three isolates, identified as strain-2, closely clustered (mean distance of 8 loci) in a different branch, having a common node with the isolate identified as strain-5; mean distance 28 loci. Strain-1 and strain-4 showed a mean distance of 120 loci from strain-2 and strain-5. The isolates identified as strain-6 and strain-7, were located in two different branches, far from the main group (mean distance of 121 and 75 loci, respectively).

Neighbour-net phylogenetic network based on a comparison of 1,325 core genome loci (cgMLST) among genomes of 22 *Neisseria meningitidis*/strain-1; three *N. meningitidis*/strain-2; two *N. meningitidis*/strain-4; one *N. meningitidis*/strain-5; one *N. meningitidis*/strain-6; one *N. meningitidis*/strain-7.

The scale bar indicates the number of allelic differences among the compared loci.

Strains 3 and 8 are not shown because they originated from three clinical samples, unsuitable for genomic analysis.

### Risk factors description

Overall, 61 of the 62 MenC cases notified during 2015–16, were investigated; one case did not participate in the face-to-face interview.

Concerning, sexual behaviours (five children < 10 years excluded from this description), among 10 cases (10/56; 17.9%) self-identified as gay-men with MSM-behaviour, five had had sexual-intercourse in the 10 days before symptom onset. Three cases (3/56; 5.3%) self-identified as bisexuals with bisexual-behaviour; two of them were women and one was a man who also reported a same-sex intercourse in the 10 days before symptoms onset.

Forty-three cases (43/56; 76.8%) reported exclusive heterosexual behaviour; and five of them had a heterosexual intercourse in the 10 days before symptom onset.

Fourteen cases (14/56; 25.0%), of whom five heterosexuals, seven MSMs and two bisexuals, attended a gay-venue in the 10 days before symptom onset. Eighteen cases (18/56; 32.1%) reported illicit drug use (7 marijuana, 11 marijuana and cocaine). Three cases (3/56; 5.3%) had clinical risk factors for IMD: two a chronic hepatitis C virus infection and one (MSM) a chronic hepatitis B virus infection. None of the cases and/or their close contacts reported travel abroad in the month before IMD onset.

### Differences in the risk factors distribution by sexual behaviour/orientation, age group and sex

At the univariate and multivariable analyses (Table), the odds of attending disco (OR: 4.51; 95% CI: 2.16–7.43; p = 0.02), sharing drinks (OR: 4.52; 95% CI: 1.18–8.34; p = 0.02) and having a sexual intercourse in the 10 days before symptom onset (OR: 3.83; 95% CI: 1.38–7.98; p = 0.01) were statistically significant higher among MSM/bisexual cases, as well as the odds of illicit drug use (AOR: 4.56; 95% CI: 2.34–8.57; p < 0.01). The odds of attending gay-venues in the 10 days before symptom onset were significantly higher among male (AOR: 4.12; 95% CI: 3.01–8.67; p < 0.01) and MSM/bisexual cases (AOR: 6.23; 95% CI: 3.25–10.1; p < 0.01).

**Table:** Crude and adjusted odds ratios of having a recognised risk factor for invasive meningococcal disease (IMD), by sexual behaviour/orientation, age group and sex among the IMD serogroup C outbreak-cases aged 11–70 years, Tuscany, Italy, 2015–2016 (n = 52)^a^

IMD risk factor		Age group in years				Sex		Sexual-behaviour/orientation	
		11–25 (20 cases)	26–40 (16 cases)	41–55 (6 cases)	56–70 (10 cases)	Female (28 cases)	Male (24 cases)	Heterosexual (39 cases)	MSM/bisexual (13 cases)
Active smoking^b^	Yes; N (%)	14 (70.0)	12 (75.0)	5 (83.3)	7 (70.0)	21 (75.0)	17 (70.8)	26 (66.7)	12 (92.3)
	No; N (%)	6 (30.0)	4 (25.0)	1 (16.7)	3 (30.0)	7 (25.0)	7 (29.2)	13 (33.3)	1 (7.7)
	OR (95% CI)	Ref	1.28 (0.20–3-45)	2.14 (0.12–3.21)	1.10 (0.15–4.12)	Ref	0.80 (0.23–2.76)	Ref	3.12 (0.15–7.21)
	p-value	Ref	0.74	0.52	0.40	Ref	0.73	Ref	0.11
Passive smoking^b^	Yes; N (%)	6 (30.0)	3 (18.7)	0 (0.0)	1 (10.0)	4 (14.3)	6 (25.0)	7 (17.9)	3 (23.1)
	No; N (%)	14 (70.0)	13 (81.3)	6 (100)	9 (90.0)	24 (85.7)	18 (75.0)	32 (82.1)	10 (76.9)
	OR (95% CI)	Ref	1.28 (0.29–5.56)	N.E.	1..34 (0.21–5.24)	Ref	2.13 (0.49–4.56)	Ref	1.37 (0.68–4.12)
	p-value	Ref	0.74	N.E.	0.64	Ref	0.33	Ref	0.54
Illicit drug use	Yes; N (%)	8 (40.0)	8 (50.0)	1 (16.7)	1 (10.0)	5 (17.9)	13 (54.2)	8 (20.5)	10 (76.9)
	No; N (%)	12 (60.0)	8 (50.0)	5 (83.3)	9 (90.0)	23 (82.1)	11 (45.8)	31 (79.5)	3 (23.1)
	OR (95% CI)	Ref	1.50 (0.39–5.65)	0.31 (0.02–3.07)	0.16 (0.17–1.58)	Ref	5.43 (1.54–7.10)	Ref	6.91 (2.34–10.13)
	p-value	Ref	0.54	0.31	0.11	Ref	< 0.01	Ref	< 0.01
	AOR (95%CI)	N.I.	N.I.	N.I.	N.I.	Ref	3.41 (0.84–7.65)	Ref	4.56 (2.34–8.57)
	p-value	N.I.	N.I.	N.I.	N.I.	Ref	0.08	Ref	< 0.01
Disco attendance^b,c^	Yes; N (%)	11 (55.0)	8 (50.0)	1 (16.7)	0 (0.0)	8 (28.6)	12 (50.0)	14 (35.9)	6 (46.1)
	No; N (%)	9 (45.0)	8 (50.0)	5 (83.3)	10 (100)	10 (71.4)	12 (50.0)	25 (64.1)	7 (53.9)
	OR (95% CI)	Ref	3.31 (0.47–5.96)	0.10 (0.01–1.11)	N.E.	Ref	1.80 (0.59–5.47)	Ref	4.51 (2.16–7.43)
	p-value	Ref	0.36	0.09	N.E.	Ref	0.30	Ref	0.02
Gay-venues attendance^c^	Yes; N (%)	8 (40.0)	6 (37.5)	0 (0.0)	0 (0.0)	2 (7.2)	12 (50.0)	5 (12.8)	9 (69.2)
	No; N (%)	12 (60.0)	10 (62.5)	6 (100)	10 (100)	26 (92.8)	12 (50.0)	34 (87.2)	4 (30.8)
	OR (95% CI)	Ref	0.77 (0.17–3.42)	N.E.	N.E.	Ref	8.47 (4.86–15.73)	Ref	9.34 (3.56–17.4)
	p-value	Ref	0.74	N.E.	N.E.	Ref	0.01	Ref	< 0.01
	AOR (95%CI)	N.I.	N.I.	N.E.	N.E.	Ref	4.12 (3.01–8.67)	Ref	6.23 (3.25–10.1)
	p-value	N.I.	N.I.	N.E.	N.E.	Ref	< 0.01	Ref	< 0.01
Bar/restaurants attendance^b,c^	Yes; N (%)	15 (75.0)	12 (75.0)	6 (100)	7 (70.0)	21 (75.0)	19 (79.2)	28 (71.8)	12 (92.3)
	No; N (%)	5 (25.0)	4 (25.0)	0 (0.0)	3 (30.0)	7 (25.0)	5 (20.8)	11 (28.2)	1 (7.7)
	OR (95% CI)	Ref	1.11 (0.21–4.56)	N.E.	0.77 (0.14–4.21)	Ref	1.26 (0.34–4.66)	Ref	5.33 (0.56–9.87)
	p-value	Ref	0.49	N.E.	0.77	Ref	0.72	Ref	0.12
Sharing drinks^b,c^	Yes; N (%)	8 (40.0)	5 (31.3)	1 (16.7)	1 (10.0)	8 (28.6)	7 (29.2)	8 (20.5)	7 (53.8)
	No; N (%)	12 (60.0)	11 (68.7)	5 (83.3)	9 (90.0)	20 (71.4)	17 (70.8)	31 (79.5)	6 (46.2)
	OR (95% CI)	Ref	0.68 (0.17–2.73)	0.30 (0.02–3.07)	0.16 (0.01 -1.58)	Ref	1.02 (0.30–3.42)	Ref	4.52 (1.18–8.34)
	p-value	Ref	0.58	0.31	0.11	Ref	0.96	Ref	0.02
Clinical conditions^b^	Yes; N (%)	0 (0.0)	1 (6.3)	0 (0.0)	2 (20.0)	1 (3.6)	2 (8.3)	2 (5.1)	1 (7.7)
	No; N (%)	20 (100)	15 (93.7)	6 (100)	8 (80.0)	27 (96.4)	22 (91.7)	37 (94.9)	12 (92.3)
	OR (95% CI)	Ref	0.26 (0.15–3.21)	N.E.	0.34 (0.20–4.67)	Ref	2.54 (0.54–7.32)	Ref	1.54 (0.67–6.78)
	p-value	Ref	0.31	N.E.	0.56	Ref	0.47	Ref	0.73
Sexual intercourse^b,c^	Yes; N (%)	3 (15.0)	5 (31.3)	1 (16.7)	2 (20.0)	5 (17.9)	6 (25.0)	5 (12.8)	6 (46.2)
	No; N (%)	17 (85.0)	11 (68.7)	5 (83.3)	8 (80.0)	23 (82.1)	18 (75.0)	34 (87.2)	7 (53.8)
	OR (95% CI)	Ref	1.88 (0.35–10.02)	1.13 (0.09–13.44)	1.14 (0.19–10.22)	Ref	1.21 (0.82–5.31)	Ref	3.83 (1.38–7.98)
	p-value	Ref	0.45	0.92	0.73	Ref	0.78	Ref	0.01

AOR: adjusted odds ratios; IMD: invasive meningococcal disease; N.E.: not estimable; N.I.: not included; OR: odds ratio; Ref: reference value.

^a^ Five cases aged under 10 years and four over 70 years were excluded from this analysis, plus one case who did not participate in the face-to-face interview.

^b^ Multivariable analysis not performed because p > 0.05 at the univariate analysis.

^c^ In the 10 days before symptom onset.

We also repeated the previous analyses including only men. Results, although based on small numbers were substantially in agreement (data not shown).

### Clusters description

**Figure 3:**
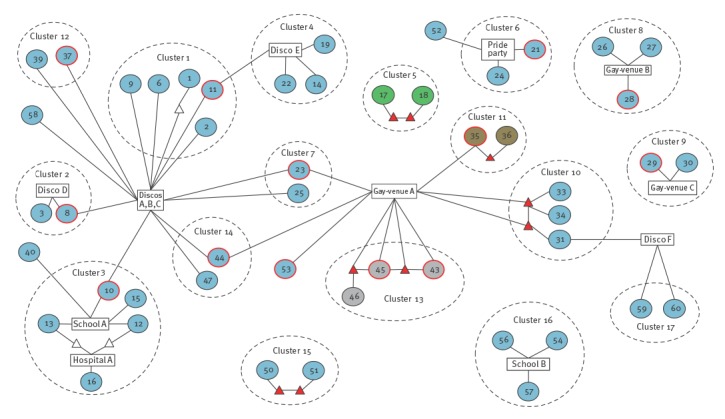
Social network diagram showing epidemiological links between serogroup C meningococcal disease cases, contacts, places and interconnections between clusters, Tuscany, 2015–2016 (n = 49)^a^

Figure 3 outlines the epidemiological links between MenC cases that emerged from the EFI. No secondary and/or co-primary cases were identified.

MSM: men who have sex with men.

Cases are numbered in chronological order of occurrence (from 1 to 62) and reported inside circles. Cases number 4, 5, 7, 20, 32, 38, 41, 42, 48, 49, 55, 61, 62 are not shown because no epidemiological links were found. Circles with blue background (n = 42) represent cases due to C:P1.5-1,10-8:F3-6:ST-11 (cc11) (Neis0430/penA 398/248) (porB 2-2) (fHbp 1.13) (ET-15) [strain 1]. Circles with grey background (n = 3) represent cases due to C:P1.5-1,10-8:F3-6:ST-11/12051 (cc11) (Neis0430/penA 6/7) (porB 2-2) (fHbp 1.808) (ET-15) [strain 2]. Circles with brown background (n = 2) represent cases due to C:P1.5-1,10-1:F3-6:ST-11 (cc11) (Neis0430/penA 398/248) (porB 2-2) (fHbp 1.13) (ET-15) [strain 3]. Circles with green background (n = 2) represent cases due to C:P1.5-1,10-8:F3-6:ST-2780 (cc11) (Neis0430/penA 398/248) (porB 2-2) (fHbp 1.13) (ET-15) [strain 4]. Circles with round solid shape outlines represent heterosexual cases (n = 36). Circles with round red solid shape outlines represents MSM/bisexual cases (n = 13). Triangles with white background (n = 3) represent heterosexual close contacts. Triangles with red background (n = 9) represent MSM close contacts. Rectangles (n = 11) represent places sorted alphabetically. Cases inside circles with long dash outlines represents clusters (n = 17).

^a ^13 casesare not shown because no epidemiological links were found.

Overall, 17 clusters involving 49 cases (80.3%) were identified. Fourteen clusters (1,2,3,4,6,7,8,9,10,12,14,15,16,17) were due to strain-1, for a total of 38 clustered and four isolated cases. Clusters 5 (two cases), 11 (two cases) and 13 (three cases) were due to the strains 4, 3 and 2 respectively.

A total of 12 close contacts acting as bridge between cases, were identified.

Six discos, four gay-venues (including a pride party), two schools (one primary and one secondary) and one hospital unit were identified as places of interconnections between cases.

Discos A,B,C, located in the same compound in the city of Florence, interconnected six clusters (1,2,3,7,12,14). Cluster-1, occurring during January–March 2015, involved five cases (1,2,6,9,11). Cases 2,6,9 attended discos A,B,C, in the 10 days before symptoms onset and a family member of case 1 worked there. Case 11 (MSM), frequented discos A,B,C and another disco (E) located in the province of Arezzo in the 10 days before symptoms onset, connecting cluster-1 to cluster-4, that involved three cases (14,19,22) during April–June 2015 who frequented disco-E in the 10 days before symptoms onset.

Cluster-2, occurring between January–February 2015, involved case 8 (MSM), who attended discos A,B,C and disco-D (located in the province of Pisa), also attended by case 3, in 10 days before symptoms onset.

Cluster-3, occurring near the city of Empoli between February–April 2015, involved five cases (10,12,13,15,16). Case 10 (MSM), attended discos A,B,C in 10 days before symptoms onset and frequented school-A, also frequented by cases 12,13,15. Two family-members of cases 12,13 worked in the same hospital unit (hospital-A) where case 16 also worked. An isolated case (40) occurring in February 2016 was connected to cluster-3 because this case frequented school-A.

Cluster-12 involved cases 37 (MSM) and 39 during January–February 2016 who frequented discos A,B,C in the 10 days before symptom onset. An isolated case (58) occurring in November 2016, also frequented discos A,B,C in the 10 days before symptom onset.

Clusters 7 and 14 worked as bridges between the clusters related to discos A,B,C and clusters 10,11,13 that had a common convergence in gay-venue-A. Cluster-7, occurring during July–September 2015, involved case 23 (MSM) who attended gay-venue-A and discos A,B,C, also attended by case 25, in 10 days before symptom onset.

Cluster-14, occurring between February–March 2016, involved two cases (44,47) who attended discos A,B,C in the 10 days before symptom onset. Case 44, was a MSM who, in the 10 days before symptom onset also attended gay-venue-A.

An isolated MSM-case (53) occurring in July 2016, also frequented gay-venue-A in the 10 days before symptom onset.

Cluster-10, occurring between December 2015–January 2016 in the province of Prato, involved cases 31,33,34. Case 33 shared the apartment with a MSM who was a family-member of case 34. This MSM had a sexual relation with another MSM who shared the apartment with case 31. These two MSM-close-contacts also attended gay-venue-A.

Cluster-10 was connected to cluster-17 (cases 59,60), occurring during November–December 2016 in the Province of Prato, through disco-F, that was frequented both by cases 31,59,60 in the 10 days before symptom onset.

Other two clusters (11,13), due to different strains (3 and 2 respectively), showed a connection with clusters 7,10,14 through gay-venue-A. Cluster-11, occurring in January 2016 in the province of Pistoia, involved cases 35 (bisexual-man) and 36. In the 10 days before symptom onset, case 35 attended gay-venue-A, and also had a sexual intercourse with a MSM-family-member of case 36.

Cluster-13, occurring in the same town of cluster-3 during February–March 2016, involved three cases (43,45,46). Cases 43,45 (MSMs) had a common MSM sexual-partner in the 10 days before symptom onset. Moreover, in the 10 days before symptom onset, case 45 also had a sexual intercourse with a MSM-family-member of case 46. Cases 43,45 and their MSM-contacts also attended gay-venue-A in the 10 days before symptom onset.

Five other isolated clusters, four (6,8,9,15,16) due to strain-1 and one (5) due to strain-4, were identified. Cluster-5, occurring during April–May 2015 in the province of Florence, involved cases 17,18 who had two MSM-family-members having a sexual relation.

Cluster-6 is represented by two cases (21, a bisexual woman – and 24) occurring during June–July 2015 who attended the Toscana Pride Party Tour in the 10 days before symptom onset, also attended by an isolated case (52) in June 2016.

Cluster-8, occurring between September–October 2015 in the province of Pisa, involved cases 28 (MSM), 26,27 who attended gay-venue-B in the 10 days before symptom onset.

Cluster-9, occurring during October–November 2015 in the province of Lucca, involved case 29 (a bisexual woman) and case 30, who attended gay-venue-C in the 10 days before symptom onset.

Cases 50,51 (cluster-15, province of Lucca) were two elderly persons with symptom onset between May–June 2016. Case 50 had a MSM-family-member who had a sexual relation with a MSM-family member of case 51.

Cluster-16, occurring between September–November 2016 in the province of Pisa, involved cases 54,56,57 who frequented school-B.

## Discussion

The increased incidence of MenC ST-11(cc11) reported in Tuscany since 2015 [6], appears to be due to a series of interconnected clusters leading to a general picture of raised incidence. Most cases were due to a clonal expansion of strain-1 in a specific area of Tuscany (so-called ‘metropolitan’), that has the highest population density (330 inhabitants per square kilometre), and where several discos and gay-venues are located [28].

MSM and bisexuals were involved in the outbreak, but IMD cases were also found among heterosexual individuals attending gay-venues. Moreover, MSM-contacts acting as bridges between cases were identified; in particular: MSM-contacts who were sexual partners of two MSM cases (e.g. cluster-13); MSM-contacts who were sexual partners of a MSM-case and close contacts (e.g. family-members) of a heterosexual case (e.g. clusters 11,13); MSM-contacts of heterosexual cases having sexual relation with MSM-contacts of other heterosexual cases (e.g. clusters 5,10,15).

This finding may be suggestive of sexual transmission of meningococci between MSM-cases/contacts with spillover of the infection to the general population (e.g. clusters 5,15), as reported in the literature [29]. However, MSM also represent a group with high nasopharyngeal carriage-rate [30], thus the sexual intercourse could be only a proxy of prolonged close contact. In addition, the occurrence of cases among heterosexual persons attending gay-venues, as well as the fact that MSM-sexual-partners of MSM-cases were also close contacts of heterosexual cases, strongly suggests that the transmission of the hyper-virulent/transmissible MenC/ST-11(cc11) strain was mainly airborne and that spillover events may have occurred [1].

MSM have been identified as a risk-group for IMD during several MenC/ST-11(cc11) outbreaks [7-11,31]. This may be partially explained by the high prevalence of risk-behaviours that facilitate close contacts with infected individuals, promoting the spread of meningococci [32,33]. In line with these findings, in our analyses MSM/bisexual cases were more prone to using illicit drugs, sharing drinks, attending discos, and having sexual intercourses (a proxy for close contact) in the 10 days before symptom onset.

No epidemiological links with the recent clusters among gay, bisexual and other MSM reported in EU/US [7-11,31] were identified. The strains isolated among MSM/bisexual cases and heterosexual cases attending gay-venues and/or having MSM-close-contacts acting as bridges, however, showed close molecular relationship with these clusters, suggesting a possible international spread of the serogroup-C-variant P1.5–1,10–8:F3–6:ST-11(cc11) among this population-group [9,11,18].

Results that emerged from the cgMLST were aligned with those that emerged from the EFI. Cases due to strains 6,7 were molecularly far from the main group and also resulted isolated, without epidemiological links at the EFI. Cases belonging to strain-2 constituted a separate cluster, showing both high genomic-similarity and epidemiological links (cluster-13); while the isolated case belonging to strain-5 clustered in a close but different branch and was not involved in any clusters at the EFI. In addition cgMLST indicated the genetic-proximity of strains 1 and 4, although at the EFI, cases due to strain-4 (17,18; cluster-5) were not connected with cases due to strain-1. Strain 1 and 4 had different ST (11 and 2,780 respectively), but were closely related according to the phylogenetic analysis, suggesting the differentiation of strain-4 from strain-1 during the outbreak-period, due to the high genomic plasticity of meningococci [34].

Discos and gay-venues were likely to play an important role as places of transmission. In this respect, outbreaks of MenC related to discos/gay-venues attendance are reported in literature [7-11,31,35,36]. According to these studies, in addition to the crowded conditions, several behavioural risk factors may converge and cumulate in these places, from smoke to drug and alcohol use, drink and cigarette sharing, as well as intimate kissing with multiple partners.

Most cases attended a bar and/or a restaurant during 10 days before symptom onset. Bar/restaurants attendance and patronage is reported in the literature as a risk factor for IMD [20]. Even if most cases attended these places, at the social network analysis they were not involved in clusters. Instead, two schools were involved in two different clusters; school-based clusters of IMD are common since meningococcal carriage is frequent in school-age groups [37].

Nosocomial transmission of *N. meningitidis* is also reported in the literature [38]. In our investigation, a hospital unit was found to be a possible transmission-hotspot; interestingly, close family members of two cases attending school A (12,13) worked in the same hospital-unit where a MenC case also worked. However, the chronological order of cases’ occurrence, and the fact that they were connected through close contacts, did not allow tracking the transmission path and defining this as nosocomial cluster.

Following case-notification, close contacts were traced and offered post-exposure prophylaxis as well as MCC/ACWY [19]. In particular, as a consequence of the notifications of 20 cases with a history of disco-attendance in the 10 days before symptom onset, more than five-thousand attendees were traced. Despite these interventions, cases related to discos (e.g. A,B,C) continued to occur. Long-term persistence of discos-associated IMD clusters due to MenC is reported in the literature [36] and discos are known to be amplifiers of *N. meningitidis* transmission [35,36]. In such context, a hyper-virulent/transmissible strain (as MenC/ST-11/cc11) is likely to have been transmitted to second-ring-contacts before the administration of prophylaxis, or might have also been reacquired after the effect of prophylaxis ended [1].

Because thousands of contacts were traced, one limitation of this investigation is that we interviewed only those close contacts who were in the family circle of cases and/or shared the same house and so other types of contacts might have been missed. This limitation implies that the epidemiological links identified could represent only the tip of the iceberg, with a pathogen that spreads silently through asymptomatic carriage [1] (in particular in the presence of the hyper-transmissible ST11/cc11 strain), and there may be other unidentified social-networks connecting the cases.

Other limits of this investigation are represented by possible recall biases, by the fact that we could interview only the close contacts of the 13 patients who died, and that incomplete information could have been provided on sensitive issues.

Moreover, the cgMLST analysis was performed only on the 30 available bacterial isolates received at ISS, allowing neither a whole genome-comparison between the 62 MenC cases investigated, nor its whole use for cluster confirmation.

Despite these limitations, risk groups, clusters and transmission-hotspots were identified. Furthermore, the combination of molecular typing and field epidemiological data, allowed the identification of distinguished clusters determined by specific molecular types of MenC over short periods of time.

## Conclusions and public health response

We described the dynamics of a multifactorial MenC outbreak characterised by a partial involvement of MSM/bisexual people; heterosexual cases attending gay-venues and MSM-close-contacts acting as bridge were also identified, suggesting a wider involvement of the MSM/bisexual community and spillover of infection to the general population.

Several clusters were recognised with transmission-hotspots in discos and gay-venues; however, two schools and one hospital-unit were also involved in three different clusters.

No epidemiological links with the recent clusters among gay, bisexual and other MSM that occurred in the EU/US were identified, but the close molecular relationship suggested a possible international spread of the serogroup-C-variant P1.5–1,10–8:F3–6:ST-11(cc11) among this population-group.

The molecular analysis allowed the confirmation of well-defined clusters due to different MenC molecular types, highlighting the spread of strain-1. A targeted vaccination campaign involving discos and LGBT associations was implemented in December 2016, and MenC cases dropped to 10 in 2017, suggesting the effectiveness of the interventions. The Italian MoH established a national enhanced surveillance and the ISS is currently monitoring the situation in the other Italian Regions; so far, strain-1 spread beyond the affected area of Tuscany has not been documented.
